# Paired Remote Ischemic Preconditioning in Recipients and Living Donors Can Mitigate Cardiovascular Stress in Recipients After Living-Donor Kidney Transplantation: A Propensity-Score-Matching Analysis

**DOI:** 10.3390/medicina60111826

**Published:** 2024-11-07

**Authors:** Jaewon Huh, Min Suk Chae

**Affiliations:** Department of Anesthesiology and Pain Medicine, Seoul St. Mary’s Hospital, College of Medicine, The Catholic University of Korea, Seoul 06591, Republic of Korea

**Keywords:** remote ischemic preconditioning, high-sensitivity troponin I, B-type natriuretic peptide, corrected QT intervals, living-donor kidney transplantation

## Abstract

*Background and Objectives*: This study explored the effect of paired remote ischemic preconditioning (RIPC), involving both recipients and living donors, on cardiovascular stress in recipients after living-donor kidney transplantation (LDKT). The analysis included an assessment of the impact on cardiovascular biomarkers and post-transplant cardiovascular clinical events. *Materials and Methods*: A retrospective observational cohort study of 520 adult LDKT patients was conducted, employing propensity score matching (PSM) to analyze perioperative factors. The patients were allocated to no-RIPC (n = 260) and paired-RIPC (n = 260) groups. The two groups were compared with respect to high-sensitivity troponin I (hsTnI) and B-type natriuretic peptide (BNP) levels, corrected QT (QTc) intervals, the occurrence of arrhythmia, and the requirement for cardiovascular interventions. *Results*: After PSM, there were no significant differences in perioperative parameters between the no-RIPC and paired-RIPC groups. However, on postoperative day (POD) 1, higher hsTnI levels and QTc interval prolongation, as well as higher incidences of arrhythmia and the need for percutaneous coronary intervention (PCI), were determined in the no-RIPC group than in the paired-RIPC group. The associations between paired RIPC and improved cardiovascular outcomes were significant, including reduced odds of elevated hsTnI levels, QTc prolongation, and arrhythmia. The no-RIPC group also had longer intensive care unit (ICU) stays, and higher rates of rescue dialysis. *Conclusions*: Paired-RIPC involving recipients and donors effectively reduces cardiovascular stress markers and improves postoperative cardiovascular outcomes in LDKT recipients, underscoring its potential as a protective strategy against perioperative cardiovascular risks.

## 1. Introduction

Patients with end-stage renal disease (ESRD) are at significantly high risk of cardiovascular events and generally have a lower quality of life [1]. Although many patients with ESRD may not show symptoms, studies indicate that 37–65% have severe coronary artery stenosis (defined as >50% blockage), which places them at high risk of both immediate and long-term cardiovascular complications or even death [2]. Kidney transplantation (KT) is recommended for patients with ESRD to reduce cardiovascular risk and enhance quality of life [3]. However, while KT has been linked to a reduction in overall long-term mortality, short-term cardiovascular mortality post-transplant increases, including a higher risk of type I myocardial infarction (MI) [4]. The latter has been attributed to plaque rupture from changes in shear stress, vasospasm, and thrombocyte activation. The risk of type II MI, which can arise from blood loss, anemia, tachycardia, or hypotension, is also higher [5]. Implementing cardiac screening and pre-/intra-transplant treatments could help mitigate the risk of postoperative cardiovascular events [6]. Nonetheless, there is no clear consensus on the best methods to improve cardiovascular outcomes in these patients.

Remote ischemic preconditioning (RIPC) is a promising strategy to shield various organs, including the heart and kidneys, from ischemic reperfusion injury [7]. Although the exact mechanisms underlying RIPC’s protective effects are still being unraveled, three primary pathways, involving humoral, neural, and systemic responses, have been implicated [8]. The efficacy of RIPC is linked to the release of autacoids, such as nitric oxide or nitrite, into the bloodstream [9], and to other signaling molecules, including endocannabinoids, stromal cell-derived factor-1α, and microRNA-144 [10]. These molecules initiate a signaling cascade that activates protein kinase and nuclear factor kappa B, leading to the production of manganese superoxide dismutase, inducible nitric oxide synthase, and other protective proteins [11]. The result is a reduction in endothelial dysfunction, better control of reactive oxygen species and proinflammatory mediators after reperfusion, and decreased cell death [12]. In patients with kidney disease, the anti-inflammatory effects of RIPC hint at its potential to protect the remaining kidney from further damage [13]. In the liver transplantation setting, ischemic preconditioning of donor organs has been shown to reduce ischemia–reperfusion injury and to enhance outcomes, indicating the broad applicability of RIPC in transplant medicine [14]. This broad potential is further supported by evidence in kidney transplantation, where early remote ischemic preconditioning of recipients has been shown to reduce ischemia–reperfusion injury and improve graft survival and renal function, underscoring RIPC as a promising protective strategy in various forms of organ transplantation [15].

In the context of KT, ischemia–reperfusion injury remains a significant cause of early graft dysfunction and contributes to long-term graft failure. Several studies have explored the potential benefits of RIPC in mitigating these effects. For example, RIPC has been shown to improve renal function in animal models of KT, where it improved glomerular filtration rate and renal plasma perfusion [16]. In clinical settings, however, the evidence is still evolving, with some studies suggesting that RIPC may reduce the incidence of delayed graft function and improve renal recovery, while others have shown limited effects [17]. Despite these mixed results, the potential for RIPC to improve transplant outcomes, particularly in LDKT, remains a compelling area of investigation.

Whether paired RIPC, involving both the recipient and the living donor, can reduce ischemic reperfusion injury of the heart in patients vulnerable to uremia is unclear. We hypothesized that paired RIPC could mitigate the decline in cardiovascular biomarkers (such as high-sensitivity troponin I [hsTnI] and B-type natriuretic peptide [BNP]) and reduce the incidence of electrocardiographic arrhythmias in recipients after living-donor kidney transplantation (LDKT). Our study included an evaluation of the influence of paired RIPC on the occurrence of post-transplant cardiovascular clinical events in recipients following LDKT.

## 2. Patients and Methods

### 2.1. Ethical Considerations

This retrospective observational cohort study was approved by the Institutional Review Board and Ethics Committee of Seoul St. Mary’s Hospital under the identifier KC22RISI0395 on 7 June 2022. This study was conducted in compliance with the ethical standards laid out in the Declaration of Helsinki. Due to its retrospective design, the requirement for informed consent from participants was waived by the ethics committee. This study is registered on ClinicalTrials.gov under the identifier NCT06280898 as of 28 February 2024. The reporting of this study adheres to the guidelines outlined in the STROBE Statement.

### 2.2. Study Population

This study initially included 640 adult patients (≥19 years of age) who underwent elective LDKT at our hospital between May 2019 and June 2023. The exclusion criteria were as follows: pediatric patients (<19 years of age), patients with arteriovenous fistulas (AVFs) that precluded RIPC on the arm, patients with arrhythmias (atrial fibrillation [Afib] or ventricular premature complexes [VPCs]) identified on the preoperative electrocardiogram (ECG), recipients with a history of significant cardiovascular diseases, including MI, coronary artery bypass grafting (CABG), heart failure, or prior percutaneous coronary intervention (PCI). Additionally, recipients of deceased-donor or ABO-incompatible kidney transplants, patients undergoing multi-organ transplants including the kidney, patients undergoing re-transplantation due to the need for diverse or complex immunosuppression regimens or surgical techniques, and those with incomplete or missing data on the recipient or donor graft were excluded from the study.

The paired-RIPC intervention was alternated based on the sequence of surgery. The decision to administer paired RIPC to both the recipient and the living donor was influenced by logistical factors, such as the availability of equipment and the surgical team’s preference, rather than specific clinical criteria. Paired-RIPC was not part of a standardized protocol during the study period, and its application varied across cases. This retrospective study utilized propensity score matching (PSM) to account for potential confounding factors and to ensure comparability between the paired-RIPC and non-RIPC groups.

**Figure 1:**
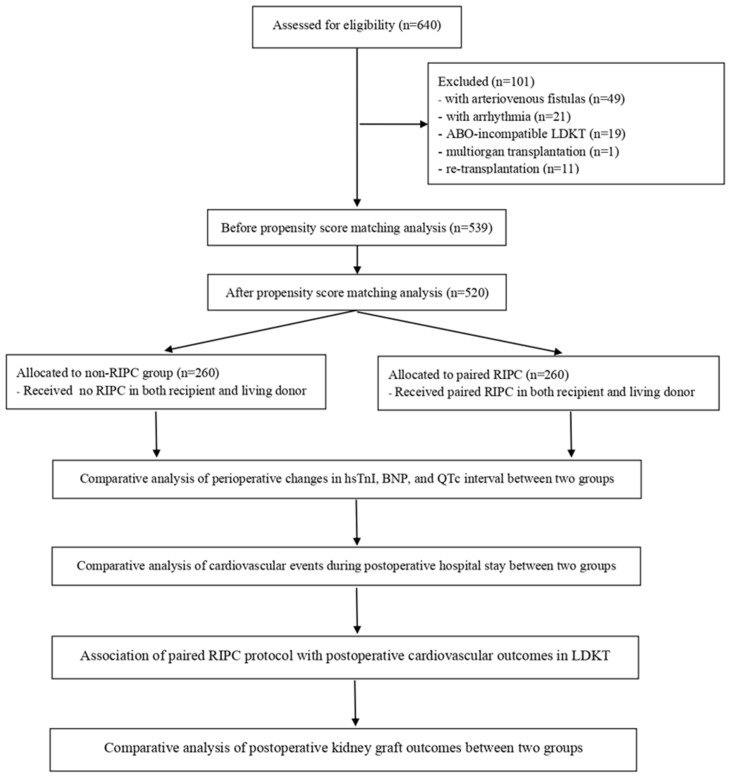
Study flow diagram.

Based on these exclusion criteria, 101 patients were deemed ineligible for this study. The data of the remaining 539 eligible patients were subjected to PSM. From the 520 matched patients, two groups were established according to whether the paired-RIPC protocol (involving both recipients and living donors) was applied: a paired-RIPC group (n = 260) and a no-RIPC group (n = 260) (Figure 1).

### 2.3. LDKT and General Anesthesia

The surgical procedure commenced with a pararectal hockey-stick-shaped (inverted J-shaped) incision to access the right pelvic fossa. Donor kidneys were preserved using standard cold ischemic techniques. Immediately post-retrieval, the kidneys were flushed and stored in University of Wisconsin (UW) solution, a commonly used preservation medium in kidney transplantation, and maintained on ice at 4 °C to limit ischemia–reperfusion injury until transplantation. The total ischemic time of each kidney was recorded as part of the perioperative analysis. Following graft preparation on the back table, end-to-side anastomosis was performed to connect the recipient’s external iliac artery and vein to the renal artery and vein of the graft, utilizing Prolene 6.0, a resorbable monofilament suture. Ureteroneocystostomy was then conducted using a Lich–Grègoir technique with a double-J stent to support ureteral reimplantation, a standard approach in kidney transplantation [18]. Once hemostasis was confirmed and vascular connections checked, closed drains were positioned, and the incision was closed.

Anesthesia was balanced, initiated with propofol and rocuronium, and maintained with desflurane in a mix of medical air and oxygen, accompanied by a continuous infusion of remifentanil. The bispectral index (BIS) was monitored to maintain hypnotic depth between 40 and 50. Hemodynamic stability was achieved with a mean arterial pressure of ≥65 mmHg, supported by inotropic agents (norepinephrine or dopamine) as necessary. Mannitol and furosemide were administered to promote urine output.

The immunosuppressive protocol included induction therapy with an interleukin-2 receptor antagonist (basiliximab) and a T-lymphocyte-depleting agent, rabbit anti-thymocyte globulin (thymoglobulin). Maintenance immunosuppression involved a calcineurin inhibitor (tacrolimus), mycophenolate mofetil, and corticosteroids. In cases of graft rejection, steroid pulse therapy and/or thymoglobulin rescue therapy were administered.

### 2.4. Paired-RIPC Intervention in Both Recipient and Living Donor

After anesthesia induction, paired RIPC was administered to the upper arms of both the living donor and recipient. For the donor, a standard blood pressure cuff was applied to the arm not used for routine blood pressure monitoring, and for the recipient, it was applied to an arm without vascular access, such as an AVF. The cuff was inflated for three cycles of 5 min inflation, followed by 5 min deflation. Each inflation reached 250 mmHg or 50 mmHg above the patient’s systolic blood pressure to ensure full arterial occlusion. To prevent any discomfort or pain at the cuff site from repeated high-pressure inflations, the intervention was performed immediately after anesthesia induction. The procedure was conducted prior to the surgical incision: in the donor, to protect against ischemic injury of the donated kidney during nephrectomy, and in the recipient, to reduce ischemia–reperfusion stress during graft reperfusion.

Both donor and recipient were closely monitored throughout the procedure to ensure stability, with particular attention to any signs of skin discoloration, bruising, or temporary changes in blood flow to the distal limb. For assessing blood flow after the intervention, a pulse oximeter was applied to the fingertip to confirm normal saturation levels and adequate circulation. Additionally, mild cardiovascular responses, such as transient changes in heart rate or blood pressure, were observed as part of the monitoring protocol. No significant complications were reported (Supplementary File S1).

### 2.5. Measurement of High-Sensitivity Troponin I and B-Type Natriuretic Peptide and Corrected QT Interval

The cardiac biomarkers hsTnI and brain BNP were meticulously measured at three pivotal time points by applying the standardized protocol of our institution: the day prior to surgery, 30 min after graft reperfusion, and on postoperative day (POD) 1 (Supplementary File S2). The hsTnI concentration was determined using an Atellica IM 1600 analyzer (Siemens Healthcare Diagnostics, Malvern, PA, USA), with high levels defined as ≥15 pg/mL for females and ≥36 pg/mL for males [19]. Plasma BNP levels were measured using an ADVIA Centaur CP immunoassay system (Siemens Healthcare Diagnostics), with high levels defined as ≥100 pg/mL [20]. For patients with multiple biomarker measurements, the most recent sample was analyzed.

Arrhythmias were monitored using the 12-lead ECG tracings obtained perioperatively as the basis for assessing ECG variables. ECG tracings were captured using a PageWriter TC50 cardiograph (Philips, Amsterdam, The Netherlands), with data extracted for both analysis and storage on the day before surgery and on POD 1. Corrected QT (QTc) interval prolongation was defined as ≥460 ms for females and ≥450 ms for males; these values served as the criteria for assessing potential cardiac arrhythmias [21].

hsTnI, BNP, and ECG were measured on POD 1 before the initiation of maintenance immunosuppressants. This approach was strategically chosen to mitigate any potential influence of these medications on biomarker levels and ECG findings, thus ensuring the accuracy and reliability of the data [22,23].

### 2.6. Clinical Variables

Preoperative, intraoperative, and postoperative recipient and donor graft factors were meticulously evaluated in the non-RIPC and paired-RIPC groups through PSM to ensure comparability, particularly regarding cardiovascular risk. The preoperative recipient factors included sex (female), age, body mass index (BMI), duration of dialysis, presence of diabetes mellitus (DM) and hypertension (HTN), systolic and diastolic blood pressures, heart rate, ejection fraction, left ventricular mass index, E/e’ ratio (a measure of diastolic function), white blood cell count, neutrophil and lymphocyte counts, hemoglobin, platelet count, albumin, sodium, potassium, creatinine, BNP, hsTnI, QTc interval, and hourly urine output. These variables were carefully selected to balance cardiovascular risk factors between the groups and minimize bias related to preoperative cardiovascular risk. Intraoperative factors included the duration of the operation and the hourly rate of fluid infusion. Factors related to the donor and graft comprised sex (female), age, BMI, hemoglobin levels, whether the left kidney was used for the graft, graft weight, and total ischemic time. Donors were evaluated for comparable baseline health characteristics to further control for cardiovascular risk. The postoperative clinical factors included the new occurrence of arrhythmias (specifically, Afib and VPC), the need for cardiovascular interventions (such as PCI or cardiopulmonary resuscitation [CPR]) during the hospital stay, lengths of stay in the intensive care unit (ICU) and hospital, the necessity of rescue dialysis (for electrolyte imbalance adjustment), re-operation (for postoperative bleeding control), and measurements of serum creatinine and hourly urine output on POD 7.

### 2.7. Statistical Analysis

A Shapiro–Wilk test was used to assess the normality of continuous variables. For normally distributed data, results are presented as the median and interquartile range (IQR). Categorical variables are displayed as counts and percentages. To reduce potential confounding, especially in the paired-RIPC group, propensity score matching (PSM) was applied. Propensity scores (PSs) were generated to enable one-to-one matching using greedy matching algorithms without replacement. Comparisons of perioperative factors between recipient and donor groups were conducted with a Mann–Whitney U test for continuous variables and either a Fisher’s exact test or χ^2^ test for categorical variables, depending on suitability. The impact of paired RIPC on postoperative cardiovascular outcomes was examined via multivariable logistic regression, adjusting for PSs. Outcomes are expressed as odds ratios (ORs) with 95% confidence intervals (CIs). All tests were two-sided, with statistical significance defined as a *p*-value < 0.05. Statistical analyses were performed using R software (version 2.10.1; R Foundation for Statistical Computing, Vienna, Austria) and SPSS (version 24.0; SPSS Inc., Chicago, IL, USA).

## 3. Results

### 3.1. Demographic Characteristics of Patients Undergoing LDKT

Among the 539 eligible recipients, 262 (48.6%) were females, and 277 (51.4%) were male. The average age was 49.4 (standard deviation [SD]: 11.7) years, and the average BMI was 23.2 (3.9) kg/m^2^. The prevalence of DM and HBP was 36.7% (n = 198) and 51.6% (n = 278), respectively. The average levels of hsTnI and BNP were 48.6 (135.5) pg/mL and 261.8 (603.1) pg/mL, respectively. The average QTc interval was 453.2 (31.1) ms. The average serum creatinine level was 7.8 (2.7) mg/dL, and the average hourly urine output was 19.9 (12.5) mL/h. Among the living donors, 339 (62.9%) were female, and 200 (37.1%) were male. Their average age was 48.3 (12.6) years, and their average BMI was 24.0 (3.2) kg/m^2^. The average total graft ischemic time was 57.9 (19.0) min, and the average graft weight was 181.4 (40.0) g.

### 3.2. Comparison of Perioperative Factors Before and After PSM

There were notable disparities in the preoperative findings of PS-unmatched patients in the two groups, specifically regarding DM and systolic blood pressure (Table 1). After PSM, the differences in perioperative recipient characteristics and donor graft parameters between the groups were no longer significant.

### 3.3. Perioperative Changes in High-Sensitivity Troponin I, Brain Natriuretic Peptide, and QTc Interval in PS-Matched Patients

The perioperative changes in hsTnI and BNP levels and in the QTc interval in the paired-RIPC and no-RIPC groups are presented in Table 2. Both the absolute levels of hsTnI and the proportion of elevated hsTnI levels (≥15 pg/mL for females and ≥36 pg/mL for males) on POD 1 were higher in the no-RIPC group than in the paired-RIPC group. However, the levels and proportions of elevated hsTnI in the two groups were similar both on the day before surgery and 30 min after reperfusion. The absolute levels and proportions of elevated BNP levels (≥100 pg/mL) in the two groups were similar on the day before surgery, 30 min after reperfusion, and on POD 1. By contrast, the duration of the QTc interval was longer and the proportion of patients with high prolongation (≥460 ms for females and ≥450 ms for males) was larger in the no-RIPC group than in the paired-RIPC group, despite similar preoperative values.

### 3.4. New Occurrence of Arrhythmia and Requirement for Cardiovascular Interventions Postoperatively in PS-Matched Patients

Cardiovascular events during the postoperative hospital stay in the no-RIPC and paired RIPC are reported in Table 3. Among patients with arrhythmias, the incidences of Afib and VPC were higher in the no-RIPC group than in the paired-RIPC group. Specifically, PCI was required more often in the no-RIPC group than in the paired-RIPC group, whereas the CPR requirement was similar.

### 3.5. Association of the Paired-RIPC Protocol with Postoperative Cardiovascular Outcomes in LDKT in PS-Matched Patients

The impact of the paired-RIPC protocol on postoperative cardiovascular outcomes in LDKT, with adjustments made for PSM, is shown in Table 4. Significant associations were determined between paired RIPC and improved cardiovascular outcomes on POD 1. The odds of having hsTnI levels above the threshold (≥15 pg/mL for females and ≥36 pg/mL for males) were significantly lower in the paired-RIPC group (OR: 0.403, 95% CI = 0.282–0.575, *p* < 0.001). Similarly, the odds of experiencing a prolonged QTc interval (≥460 ms for females and ≥450 ms for males) were lower in the paired-RIPC group (OR: 0.246, 95% CI = 0.156–0.39, *p* < 0.001). The incidences of arrhythmia were significantly lower in the paired-RIPC group, both for Afib (OR: 0.249, 95% CI = 0.106–0.585, *p* = 0.001) and for VPC (OR: 0.255, 95% CI = 0.119–0.547, *p* < 0.001).

### 3.6. Postoperative Kidney Graft Outcomes in PS-Matched Patients

The postoperative kidney graft outcomes of the no-RIPC and paired-RIPC patients are presented in Table 5. The no-RIPC group had a longer duration of ICU hospitalization and a significantly higher incidence of a need for rescue dialysis.

### 3.7. Comparison of Postoperative Cardiovascular Outcomes or Graft Function Between the Original and PSM Cohorts

This study used propensity score matching to balance key preoperative, perioperative, and donor-graft characteristics between the paired-RIPC and non-RIPC groups. The variables included in the propensity score model were age, sex, BMI, presence of diabetes mellitus and hypertension, baseline cardiovascular parameters (such as blood pressure and ejection fraction), laboratory values (including high-sensitivity troponin I and B-type natriuretic peptide), intraoperative factors (such as operation time and fluid administration rates), and donor-graft characteristics (such as ischemic time and graft weight).

We further analyzed the comparability between the propensity-score-matched cohort and the original cohort. There were no significant differences in the postoperative cardiovascular outcomes or graft function between the original and PSM cohorts, indicating that the findings in both cohorts are consistent. This comparability reinforces the robustness of our results, suggesting that the observed effects of paired-RIPC on cardiovascular and renal outcomes are valid across both cohorts.

## 4. Discussion

This study demonstrated that paired RIPC, involving both recipients and living donors, effectively reduced peak levels of hsTnI and mitigated QTc prolongation in recipients following LDKT. Patients in the paired-RIPC group experienced a significant reduction in new arrhythmic events, including Afib and VPCs, as well as a decreased need for PCI to manage cardiovascular complications. The likelihood of adverse cardiovascular outcomes postoperatively was markedly lower in the paired-RIPC group, with an odds ratio of 0.403 for elevated hsTnI levels, 0.246 for QTc prolongation, 0.249 for Afib, and 0.255 for VPCs. Additionally, paired RIPC was associated with improved kidney graft outcomes, including shorter ICU stays and a reduced need for rescue dialysis.

Clinical evidence from a comprehensive meta-analysis of 13 trials involving 1968 participants demonstrated that RIPC significantly reduces the risk of cardiovascular events, such as MI, cardiac arrest, and heart failure, with an odds ratio of 0.68, highlighting RIPC’s potential to improve cardiovascular outcomes in various surgical settings [24]. To minimize complications, regular risk stratification and monitoring of biomarkers such as hsTnI and BNP, along with ECG assessments, are critical for timely interventions in patients at risk of adverse cardiovascular events [25,26]. Our RIPC protocol shows substantial benefits for transplant patients at risk of cardiac dysfunction, particularly those with coronary artery disease risk factors (e.g., DM, HTN, hyperlipidemia) and hemodynamic stressors such as volume overload, anemia, uremic toxin accumulation, and disturbances in calcium–phosphorus metabolism. By targeting these diverse risk factors, our approach helps lower hsTnI levels and provides cardioprotection, safeguarding organ health [27,28,29]. A meta-analysis of 19 randomized trials involving 1235 patients further demonstrated that RIPC significantly reduces both postoperative cardiac troponin I levels at 6 h (within a 4–8 h range) and total postoperative troponin I release. The weighted mean difference in troponin I concentrations was –2.03 ug/L, with a total troponin I release reduction of –65.74 ug/L/h, indicating a substantial reduction in myocardial injury [29]. In patients undergoing cardiac surgery, including CABG, plasma troponin I levels remained significantly lower in the RIPC group compared to controls at both 6 and 24 h postoperatively [30].

The lack of effect of paired RIPC on BNP levels both intraoperatively and on POD 1 can be attributed to volume overload and circulatory stress, which may trigger an earlier release of BNP from stretched cardiac myocytes. However, improvements in graft kidney function, leading to increased urine output, may help alleviate the recipient’s volume and circulatory stress after LDKT. Elevated plasma BNP levels are commonly observed in patients with chronic renal failure, reflecting cardiac stress and dysfunction primarily due to volume overload, among other factors associated with renal failure [31]. BNP levels are also a useful marker of allograft dysfunction post-KT. Studies have documented a significant reduction in BNP levels after successful allograft transplantation, underscoring the improvement in renal function and the subsequent relief of cardiac stress. This highlights the complex relationship between renal and cardiac health, with BNP serving as a key biomarker for the interplay between renal function and cardiac status in the post-transplant period [32]. In both the paired-RIPC and non-RIPC groups, kidney grafts maintained adequate urine output, which helped mitigate increased circulatory volume and pressure overload. This reduction in volume overload likely contributed to the observed decrease in BNP levels after surgery, indicating an overall improvement in cardiac circulatory stress.

Postoperative patients frequently experience significant QTc interval prolongation, a marker of abnormal cardiac repolarization that increases the risk of torsades de pointes, a potentially fatal arrhythmia. This prolongation is often challenging to quantify but is thought to be driven by surgical stress, which is consistent with the substantial effect of epinephrine on extending the QTc interval [21]. In our study, the QTc interval increased by an average of 23 ms, with some patients experiencing increases of more than 60 ms and reaching absolute QTc intervals of 500 ms. However, this prolongation was transient, occurring only during the stay in the PACU and resolving by subsequent postoperative days [33]. Our paired-RIPC protocol may help reduce surgical stress in recipients and mitigate the immunoinflammatory responses associated with LDKT [34,35,36]. This approach has the potential to prevent QTc interval prolongation, offering a protective advantage compared to no-RIPC treatment. A study of RIPC’s effects on QT intervals during exercise in healthy individuals demonstrated its potential to reduce QTc prolongation, which is critical for cardiovascular health. In a study involving 17 participants, RIPC—consisting of four 5 min ischemia/reperfusion cycles—significantly reduced QT interval prolongation during both exercise and recovery phases without affecting heart rate. This highlights RIPC’s capacity to mitigate QTc prolongation independently of heart rate changes during physical exertion [28]. By targeting the stress and inflammatory reactions that contribute to cardiac repolarization abnormalities, our protocol may enhance postoperative recovery and protect cardiovascular health in KT patients.

Our paired-RIPC protocol shows promise in preventing myocardial repolarization abnormalities, as reflected by the QTc interval, potentially reducing the incidence of postoperative arrhythmias, including Afib and VPCs. A study by Mandyam et al. found that a prolonged QT interval was associated with a significantly higher risk of developing Afib. Each increase in the QT interval correlated with a greater risk of Afib in both unadjusted and adjusted analyses [37]. RIPC has been shown to decrease the inducibility and sustainability of Afib, likely by improving atrial electrophysiological properties, such as reducing the dispersion of atrial refractory periods and atrial conduction delays—indicators of enhanced atrial conduction [27]. Additionally, Karaman et al. found that the Tp-e interval and Tp-e/QTc ratios were significantly elevated in patients with a higher VPC burden, showing a positive correlation between these repolarization markers and VPC frequency. The worsening of myocardial repolarization with increasing VPC frequency highlights the importance of monitoring these parameters to mitigate the risk of postoperative arrhythmias [38]. These findings underline the broad therapeutic potential of RIPC in improving cardiovascular health and preventing electrophysiological complications. As a non-invasive strategy, RIPC offers a valuable approach to reducing the risk of significant postoperative cardiac events.

In our study, the favorable effects of paired-RIPC on cardiovascular outcomes, such as reductions in hsTnI levels and QTc prolongation, were not observed immediately after reperfusion but became evident by POD 1. This delay is consistent with the proposed underlying mechanism of action at the molecular level, involving the upregulation and synthesis of protective proteins such as manganese superoxide dismutase (MnSOD) and inducible nitric oxide synthase (iNOS). These proteins play critical roles in reducing oxidative stress and inflammation, which can protect the myocardium and other tissues from ischemia–reperfusion injury. The time required for the transcription, translation, and subsequent activation of these protective proteins likely accounts for the latency in the observable effects of RIPC [11,13,39]. This hypothesis is supported by the lack of significant differences between the paired-RIPC and non-RIPC groups 30 min after reperfusion, with the beneficial effects becoming apparent only on POD 1. Such a delayed response highlights the importance of timing when assessing the cardioprotective effects of RIPC.

During the postoperative period, patients in the paired-RIPC group had a lower incidence of PCI requirement than those in the non-RIPC group. Although our study did not specifically focus on PCI outcomes, the existing literature suggests that RIPC may offer cardioprotection for patients undergoing elective or emergency PCI. Studies have shown reductions in post-PCI troponin levels and infarct size, although the exact mechanisms remain incompletely understood [40,41]. In a study by Lau et al., RIPC was shown to acutely improve coronary microcirculatory function, as demonstrated by a reduction in the index of microcirculatory resistance and an increase in coronary flow reserve. These changes were accompanied by a decrease in hyperemic transit time. Compared to a sham procedure, RIPC significantly improved both microcirculatory resistance and coronary flow reserve [42]. These findings suggest that by enhancing coronary microcirculation, RIPC may reduce the need for PCI and mitigate myocardial injury in the perioperative setting.

A study focusing on LDKT showed that RIPC offers significant benefits in preserving kidney function in donors. Notably, postoperative serum creatinine levels were considerably lower in donors treated with RIPC, and this benefit was sustained for up to one year postoperatively. Recovery of the estimated glomerular filtration rate within the first month after surgery was also faster in these donors, underscoring RIPC’s pivotal role in promoting renal recovery and function preservation [43]. In our study, we observed that paired-RIPC significantly improved kidney graft function in recipients, as evidenced by a reduced need for rescue hemodialysis in the paired-RIPC group compared to the non-RIPC group. This suggests that paired RIPC may also help reduce ischemia–reperfusion injury in transplanted kidneys, enhancing graft viability and function. Despite the positive effects of RIPC in donors, some studies have not observed similar improvements in kidney function among recipients with preconditioned donor kidneys. The absence of improvement in these cases may be attributed to RIPC’s limited ability to counter ischemia–reperfusion injury, particularly in patients less prone to severe ischemic injury [44]. Interestingly, ischemic preconditioning has been shown to result in better transplant outcomes in marginal-quality liver grafts than in higher-quality grafts, suggesting that RIPC could benefit less optimal kidney grafts as well [14]. Furthermore, the efficacy of RIPC-induced protection can be influenced by various factors, including comorbidities and medications. Preclinical studies have shown that factors such as older age, hyperlipidemia, DM, and HTN, which are common among KT recipients, may increase the threshold for RIPC’s protective effects, necessitating stronger conditioning [39]. The variability in RIPC’s protective efficacy may also be affected by the degree of surgical stress, intraoperative conditions, and patient-specific characteristics [24,27,35]. As such, the effectiveness of RIPC in improving post-transplant outcomes may depend on the particular circumstances encountered during surgery, highlighting the need for a tailored approach to its application in transplantation surgeries.

This study has several important limitations that may impact its broader applicability. First, as RIPC was administered via the arm, patients who had undergone long-term dialysis through an AVF were excluded, as the AVF precludes RIPC application at that site. Given that many patients awaiting KT receive dialysis through an AVF, this limitation restricts the generalizability of our findings. Future research should aim to include these patients, potentially applying RIPC at alternative sites, such as the lower extremity, to evaluate its effects in this subgroup. Second, to minimize potential cardiac effects of immunosuppressive agents [22], this study measured blood tests and ECG results before the administration of immunosuppressants on POD 1. However, we recognize that immunosuppressive therapy can influence long-term cardiovascular outcomes, and further research is needed to assess the ongoing effects of immunosuppressant use in the postoperative period. Third, our study focused on paired RIPC between living donors and recipients, which limits its applicability to deceased-donor transplants. Evaluating the effects of paired RIPC in deceased-donor–recipient pairs would be beneficial for a more comprehensive understanding of RIPC’s potential across different transplant types. Fourth, this study evaluated short-term cardiac injury markers, such as hsTnI, BNP, and QTc interval, to assess the immediate effects of paired-RIPC. While these markers provide valuable insights into perioperative cardiac stress and injury, they do not capture long-term cardiovascular events, including the need for PCI, the development of arrhythmias, or the occurrence of Type I and Type II myocardial infarctions. Recognizing the importance of these outcomes, we plan to include extended follow-up and broader cardiovascular assessments in future research, allowing for a more comprehensive evaluation of paired RIPC’s long-term benefits and risks in KT. Lastly, despite the use of PSM to balance key baseline characteristics between the paired-RIPC and non-RIPC groups, some variations, particularly in comorbidities such as DM and HTN, persisted after matching. These differences could reflect residual confounding, a limitation common to retrospective studies. Nevertheless, our statistical analyses accounted for these variables, and they did not significantly impact the primary outcomes. A notable strength of this study is its dual focus on living donors and recipients within the context of LDKT, allowing for a unique evaluation of the cardioprotective effects of paired RIPC in recipient–living-donor pairs. These findings offer valuable insights for a specific clinical setting where both parties may benefit from the intervention.

## 5. Conclusions

This study suggests that paired-RIPC may provide short-term cardioprotective effects during the perioperative period in LDKT, as demonstrated by reductions in cardiac biomarkers such as hsTnI and improvements in QTc intervals. Additionally, while our data indicate potential benefits for kidney graft function, particularly in reducing the need for rescue hemodialysis, no strong direct correlation between RIPC and long-term cardiovascular health or kidney function was observed in this study. Although the findings from this retrospective analysis are encouraging, they warrant further investigation. Future studies, including randomized controlled trials, are necessary to confirm the clinical efficacy of paired RIPC in LDKT. Given the promising results observed in this study, a prospective proof-of-concept trial with a relatively small number of patients may be feasible to further validate the strong short-term cardioprotective effects of RIPC. Such a trial could provide crucial evidence to establish paired RIPC as a standard intervention aimed at reducing postoperative cardiovascular complications and enhancing kidney graft outcomes in transplant recipients.

## Figures and Tables

**Table 1:** Clinical perioperative findings in the no-RIPC and paired-RIPC groups before and after propensity score matching.

	Before Propensity Score Matching				After Propensity Score Matching			
Group	No-RIPC	Paired-RIPC	*p*	SD	No-RIPC	Paired-RIPC	*p*	SD
n	270	269			260	260		
Preoperative recipient factors								
Sex (female)	124 (45.9%)	138 (51.3%)	0.212	0.107	118 (45.4%)	132 (50.8%)	0.219	0.108
Age (years)	50.0 (40.0–57.0)	52.0 (41.5–59.0)	0.126	0.115	50.0 (41.0–57.0)	52.0 (42.0–59.0)	0.135	0.113
Body mass index (kg/m^2^)	22.9 (20.4–25.4)	22.8 (20.6–26.0)	0.676	0.071	23.0 (20.3–25.5)	23.0 (20.6–26.1)	0.557	0.082
Dialysis duration (day)	1.0 (0.0–11.0)	1.0 (0.0–7.5)	0.9	−0.081	0.5 (0.0–10.0)	1.0 (0.0–6.0)	0.86	−0.080
Comorbidity								
Diabetes mellitus	86 (31.9%)	112 (41.6%)	0.018	0.213	84 (32.3%)	105 (40.4%)	0.056	0.179
Hypertension	132 (48.9%)	146 (54.3%)	0.211	0.108	127 (48.8%)	142 (54.6%)	0.188	0.116
Vital sign								
Systolic blood pressure (mmHg)	130.0 (120.0–140.0)	132.0 (124.0–141.0)	0.033	0.178	130.0 (120.0–140.0)	131.0 (124.0–140.0)	0.063	0.150
Diastolic blood pressure (mmHg)	80.0 (79.0–90.0)	80.0 (80.0–90.0)	0.256	0.092	80.0 (80.0–90.0)	80.0 (80.0–90.0)	0.38	0.065
Heart rate (beats/min)	80.0 (73.0–88.0)	80.0 (73.0–88.0)	0.583	0.042	80.0 (73.0–88.0)	62.0 (58.8–64.9)	0.492	−0.021
Echocardiography								
Ejection fraction (%)	62.0 (57.3–64.6)	62.0 (58.7–64.8)	0.314	0.086	62.0 (58.0–64.7)	62.0 (58.8–64.9)	0.484	0.037
Left ventricular mass index (g/m^2^)	119.1 (102.0–144.0)	119.1 (101.0–140.3)	0.376	−0.109	119.1 (101.0–142.0)	118.7 (99.0–140.3)	0.477	−0.097
E/e’ ratio	10.0 (7.8–12.7)	10.3 (8.7–12.9)	0.17	0.005	10.0 (7.8–12.5)	10.3 (8.7–12.6)	0.142	0.008
Laboratory variables								
White blood cell count (×10^9^/L)	6.3 (4.8–7.8)	6.2 (4.8–8.1)	0.983	−0.026	6.3 (4.8–7.8)	6.2 (4.7–8.1)	0.945	−0.027
Neutrophil (%)	67.2 (60.7–82.9)	70.5 (61.6–85.4)	0.284	0.099	67.4 (60.6–82.9)	70.3 (61.5–85.2)	0.367	0.087
Lymphocyte (%)	19.7 (11.6–26.3)	18.4 (10.9–25.2)	0.211	−0.106	19.7 (11.8–26.2)	18.7 (10.9–25.1)	0.261	−0.099
Hemoglobin (g/dL)	10.6 (9.4–11.6)	10.7 (9.6–11.5)	0.508	0.025	10.6 (9.5–11.6)	10.7 (9.6–11.5)	0.649	0.016
Platelet count (×10^9^/L)	178.5 (141.0–230.3)	178.0 (140.5–218.0)	0.466	−0.114	180.0 (141.0–230.8)	178.0 (140.3–216.8)	0.333	−0.132
Albumin (g/dL)	4.1 (3.8–4.3)	4.1 (3.8–4.3)	0.54	0.053	4.1 (3.8–4.4)	4.1 (3.8–4.3)	0.868	−0.014
Sodium (mEq/L)	138.0 (135.0–140.0)	138.0 (135.0–140.0)	0.891	0.021	138.0 (135.0–140.0)	138.0 (135.0–140.0)	0.865	0.001
Potassium (mEq/L)	4.7 (4.3–5.2)	4.7 (4.2–5.2)	0.577	−0.025	4.7 (4.3–5.2)	4.7 (4.2–5.2)	0.462	−0.045
Creatinine (mg/dL)	7.6 (5.9–9.3)	7.1 (6.0–9.1)	0.371	−0.060	7.5 (5.9–9.1)	7.2 (6.0–9.1)	0.632	−0.015
B-type natriuretic peptide (pg/mL)	82.4 (36.1–218.7)	78.7 (31.6–177.2)	0.166	−0.391	76.7 (34.1–194.3)	77.0 (31.6–176.8)	0.44	−0.159
High-sensitivity troponin I (pg/mL)	20.5 (10.4–46.3)	21.4 (11.1–44.6)	0.631	−0.150	20.4 (10.4–46.3)	21.1 (11.0–44.2)	0.589	−0.057
Corrected QT interval (ms)	452.0 (432.0–475.0)	450.0 (431.0–469.5)	0.431	−0.069	452.0 (432.0–473.0)	450.0 (430.3–469.0)	0.497	−0.061
Hourly urine output (mL/kg/h)	0.3 (0.2–0.4)	0.3 (0.2–0.4)	0.162	0.131	0.3 (0.2–0.4)	0.3 (0.2–0.4)	0.242	0.108
Intraoperative recipient factors								
Operation time (min)	225.0 (190.0–260.0)	225.0 (195.0–255.0)	0.677	0.015	223.5 (190.0–260.0)	225.0 (195.0–255.0)	0.533	0.037
Hourly fluid infusion (mL/kg/h)	9.3 (7.3–11.6)	9.0 (7.4–11.4)	0.567	−0.062	9.3 (7.3–11.6)	8.9 (7.2–11.3)	0.344	−0.094
Donor and graft factors								
Sex (female)	176 (65.2%)	163 (60.6%)	0.27	−0.094	168 (64.6%)	160 (61.5%)	0.467	−0.063
Age (years)	51.0 (41.0–57.0)	51.0 (38.0–59.0)	0.941	−0.023	51.0 (41.0–57.0)	51.0 (38.0–59.0)	0.983	−0.024
Body mass index (kg/m^2^)	23.6 (21.7–25.6)	23.5 (21.9–26.1)	0.602	0.068	23.7 (21.8–25.7)	23.4 (21.9–26.1)	0.911	0.038
Hemoglobin (g/dL)	13.7 (12.8–14.9)	13.9 (12.9–15.1)	0.54	0.035	13.7 (12.8–14.9)	13.8 (12.9–15.1)	0.647	0.024
Left kidney graft	162 (60.0%)	174 (64.7%)	0.262	−0.098	156 (60.0%)	169 (65.0%)	0.239	−0.104
Graft weight (g)	178.0 (150.0–204.5)	174.0 (150.0–208.0)	0.81	−0.001	178.0 (152.0–205.5)	174.0 (150.0–208.0)	0.635	−0.017
Total ischemic time (min)	55.0 (43.8–67.3)	54.0 (45.0–67.5)	0.885	−0.041	55.0 (43.3–67.0)	54.5 (45.0–68.0)	0.899	−0.004

Abbreviations: RIPC, remote ischemic preconditioning; SD, standard deviation. Note: The dialysis duration reflects the total number of days patients required postoperative rescue hemodialysis. Values are expressed as median (interquartile) and number (percentage).

**Table 2:** Perioperative changes in high-sensitivity troponin I, B-type natriuretic peptide, and the corrected QT interval in the no-RIPC and paired-RIPC groups.

Group	No-RIPC	Paired-RIPC	*p*
n	260	260	
Cardiac enzymes			
hsTnI (pg/mL)			
Preoperative day	20.4 (10.4–46.1)	21.0 (11.0–44.2)	0.57
30 min after reperfusion	21.3 (11.5–53.4)	21.8 (12.3–47.2)	0.877
POD 1	40.2 (19.2–81.3)	21.1 (10.1–43.0)	<0.001
hsTnI ≥ 15 pg/mL for female and ≥36 pg/mL for male			
Preoperative day	119 (45.8%)	125 (48.1%)	0.598
30 min after reperfusion	127 (48.8%)	126 (48.5%)	>0.999
POD 1	176 (67.7%)	119 (45.8%)	<0.001
BNP (pg/mL)			
Preoperative day	76.7 (34.1–194.3)	77.0 (31.6–176.8)	0.44
30 min after reperfusion	164.3 (88.0–331.1)	173.2 (90.2–336.9)	0.703
POD 1	129.1 (74.2–259.6)	116.9 (79.2–227.7)	0.391
BNP ≥ 100 pg/mL			
Preoperative day	109 (41.9%)	110 (42.3%)	0.929
30 min after reperfusion	177 (68.1%)	181 (69.6%)	0.705
POD 1	162 (62.3%)	160 (61.5%)	0.857
Electrocardiogram			
QTc (ms)			
Preoperative day	452.0 (432.0–473.0)	450.0 (430.3–469.0)	0.497
POD 1	498.5 (477.0–515.0)	467.0 (444.8–492.0)	<0.001
QTc ≥ 460 ms for female and ≥450 ms for male			
Preoperative day	119 (45.8%)	103 (39.6%)	0.156
POD 1	230 (88.5%)	170 (65.4%)	<0.001

Abbreviations: QTc, corrected QT interval; BNP, B-type natriuretic peptide; POD, postoperative day. Values are expressed as median and interquartile.

**Table 3:** Cardiovascular events during the postoperative hospital stay in the no-RIPC and paired-RIPC groups.

Group	No-RIPC	Paired-RIPC	*p*
n	260	260	
New occurrence of arrhythmia			
Atrial fibrillation	26 (10.0%)	7 (2.7%)	0.001
Ventricular premature complex	32 (12.3%)	9 (3.5%)	<0.001
Requirement of cardiovascular interventions			
Percutaneous coronary intervention	12 (4.6%)	4 (1.5%)	0.042
Cardiopulmonary resuscitation	2 (0.8%)	1 (0.4%)	>0.999

Values are expressed as numbers and percentages.

**Table 4:** Association of paired-RIPC protocol with postoperative cardiovascular outcomes in living donor kidney transplantation.

	*β*	Odds Ratio	95% CI	*p*
Paired RIPC adjusted for PS				
hsTnI ≥ 15 pg/mL for female and ≥36 pg/mL for male on POD 1	−0.909	0.403	0.282–0.575	<0.001
BNP ≥ 100 pg/mL on POD 1	−0.033	0.968	0.679–1.379	0.857
QTc ≥ 460 ms for female and ≥450 ms for male on POD 1	−1.401	0.246	0.156–0.39	<0.001
New occurrence of arrhythmia during postoperative hospital stay				
Atrial fibrillation	−1.39	0.249	0.106–0.585	0.001
Ventricular premature complex	−1.365	0.255	0.119–0.547	<0.001
Requirement of cardiovascular interventions during postoperative hospital stay				
Percutaneous coronary intervention	−1.13	0.323	0.103–1.015	0.053
Cardiopulmonary resuscitation	−0.697	0.498	0.045–5.527	0.57

Abbreviations: PS, propensity score; POD, postoperative day; BNP, B-type natriuretic peptide; CI, confidence interval.

**Table 5:** Postoperative kidney graft outcomes in the no-RIPC and paired-RIPC groups.

Group	No-RIPC	Paired-RIPC	*p*
n	260	260	
Administration period (days)			
ICU stay	4.0 (2.0–5.0)	2.0 (2.0–3.0)	<0.001
hospital stay	14.0 (12.0–15.0)	13.0 (12.0–15.0)	0.351
Requirement of rescue dialysis	26 (10.0%)	13 (5.0%)	0.03
Requirement of re-operation	3 (1.2%)	3 (1.2%)	>0.999
Serum creatinine (mg/dL)			
Preoperative day	7.5 (5.9–9.1)	7.2 (6.0–9.1)	0.632
POD 1	2.7 (1.9–3.5)	2.6 (1.8–3.4)	0.438
POD 2	1.3 (1.0–1.8)	1.3 (0.9–1.9)	0.981
POD 3	1.1 (0.8–1.5)	1.1 (0.8–1.5)	0.887
POD 7	0.9 (0.7–1.2)	0.9 (0.7–1.2)	0.792
Hourly urine output (mL/kg/h)			
Preoperative day	0.3 (0.2–0.4)	0.3 (0.2–0.4)	0.242
POD 1	6.4 (5.0–8.5)	6.6 (5.0–8.4)	0.823
POD 2	4.5 (3.6–5.6)	4.6 (3.6–5.7)	0.807
POD 3	4.0 (3.3–5.1)	4.0 (3.1–5.0)	0.422
POD 7	2.2 (1.8–2.8)	2.3 (1.9–2.8)	0.103

Abbreviations: ICU, intensive care unit; POD, postoperative day. Values are expressed as median and interquartile.

## Data Availability

Data are contained within the article or Supplementary Materials.

## Supplementary Materials

The following supporting information can be downloaded at: https://www.mdpi.com/article/10.3390/medicina60111826/s1, Supplementary File S1: Paired-RIPC intervention protocol in living donor and recipient. Supplementary File S2: Measurement of high-sensitivity troponin I and B-type natriuretic peptide, and corrected QT interval in recipients.

## Abbreviations

RIPC: remote ischemic preconditioning; LDKT, living donor kidney transplantation; QTc, corrected QT; hsTnI, High-sensitivity troponin I; BNP, B-type natriuretic peptide; PCI, percutaneous coronary intervention; AVF, arteriovenous fistula; POD, postoperative day; Afib, atrial fibrillation; VPC, ventricular premature complex; PS, propensity score.
