# Supplementary material for: Paired Remote Ischemic Preconditioning in Recipients and Living Donors Can Mitigate Cardiovascular Stress in Recipients After Living-Donor Kidney Transplantation: A Propensity-Score-Matching Analysis
Source: Medicina (Kaunas). 2024 Nov 7;60(11):1826. doi: 10.3390/medicina60111826 (PMC11596797; doi:10.3390/medicina60111826)
Supplement: Supplementary file 1 [file medicina-60-01826-s001.zip › Supplementary file S1.pdf]

Supplementary File S1. Paired-RIPC intervention protocol in living donor and recipient

Aspect	Living donor	Recipient
**Timing of intervention**	Administered immediately after anesthesia induction, before surgical incision<br>- At least before vascular clamping of the donated kidney	Administered immediately after anesthesia induction, before surgical incision<br>- At least before graft reperfusion
**Cuff Placement**	Blood pressure cuff applied to the arm not used for routine blood pressure monitoring.	Blood pressure cuff applied to an arm without vascular access, such as an arteriovenous fistula
**Cycles**	3 cycles of 5-minute inflation followed by 5-minute deflation	
**Inflation Pressure**	250 mmHg or 50 mmHg above the donor's systolic blood pressure to ensure full arterial occlusion.	250 mmHg or 50 mmHg above the recipient's systolic blood pressure to ensure full arterial occlusion.
**Monitoring During Intervention**	Continuous monitoring for stability, with specific attention to<br>1) Skin discoloration,<br>2) Bruising<br>3) Signs of reduced blood flow to the distal limb.<br>4) Cardiovascular responses (e.g., transient changes in heart rate or blood pressure)	
**Post-Intervention Blood Flow Check**	Pulse oximetry applied to the fingertip to confirm adequate blood flow and normal saturation levels in the distal limb.	
