# Supplementary material for: Paired Remote Ischemic Preconditioning in Recipients and Living Donors Can Mitigate Cardiovascular Stress in Recipients After Living-Donor Kidney Transplantation: A Propensity-Score-Matching Analysis
Source: Medicina (Kaunas). 2024 Nov 7;60(11):1826. doi: 10.3390/medicina60111826 (PMC11596797; doi:10.3390/medicina60111826)
Supplement: Supplementary file 1 [file medicina-60-01826-s001.zip › Supplementary file S2.pdf]

Supplementary file S2. Measurement of high-sensitivity troponin I and B-type natriuretic peptide, and corrected QT interval in recipients

Aspect	High-Sensitivity Troponin I (hsTnI)	B-type Natriuretic Peptide (BNP)	Corrected QT (QTc) Interval
**Measurement Timing**	- Day before surgery<br>- 30 min after graft reperfusion<br>- POD 1 (before immunosuppressants)	- Day before surgery<br>- 30 min after graft reperfusion<br>- POD 1 (before immunosuppressants)	- Day before surgery<br>- POD 1 (before immunosuppressants)
**Description**	A cardiac biomarker measured to assess myocardial injury.	A biomarker measured to evaluate heart failure or cardiac stress.	ECG measurement for assessing arrhythmia risk through QT interval prolongation.
**High-Level Definition**	≥ 15 pg/mL (females)<br>≥ 36 pg/mL (males)	≥ 100 pg/mL	QTc prolongation: ≥ 460 ms (females)<br>QTc prolongation: ≥ 450 ms (males)
